# How Urinary Incontinence Affects Sexual Activity in Polish Women: Results from a Cross-Sectional Study

**DOI:** 10.3390/ijerph192113818

**Published:** 2022-10-24

**Authors:** Bartlomiej Burzynski, Piotr Gibala, Zuzanna Soltysiak-Gibala, Tomasz Jurys, Piotr Przymuszala, Pawel Rzymski, Rafal Stojko

**Affiliations:** 1 Department of Rehabilitation, Faculty of Health Sciences in Katowice, Medical University of Silesia in Katowice, Medykow 12, 40-752 Katowice, Poland; 2 Chair and Department of Gynecology, Obstetrics and Gynecological Oncology, Faculty of Health Sciences in Katowice, Medical University of Silesia in Katowice, Medykow 12, 40-752 Katowice, Poland; 3 Doctoral School, Faculty of Health Sciences in Katowice, Medical University of Silesia in Katowice, Medykow 12, 40-752 Katowice, Poland; 4 Department of Medical Education, Poznan University of Medical Sciences, Fredry 10, 61-701 Poznan, Poland; 5 Department of Mother and Child Health, Poznan University of Medical Sciences, Gynecological and Obstetric University Hospital, Polna 33, 60-535 Poznan, Poland

**Keywords:** sexual function, urinary incontinence, sexual health, quality of life

## Abstract

Urinary incontinence (UI) in women can cause a number of sexual dysfunctions and reduce their quality of life. Sexual health is essential to the self-esteem, emotional state, and overall quality of life. We aimed to assess the quality of sexual life of women with UI. The study included a group of 145 sexually active women diagnosed with UI. The research was conducted using the diagnostic survey method with authorial and validated questionnaires. As many as 49.1% of the respondents reported a deterioration of sexual relations in comparison with the time before the onset of UI symptoms. According to the FSDS-R results, 83.45% of respondents were dissatisfied with their sex life. The higher the result obtained by respondents in the FSDS-R scale, the lower was their quality of life in the IIQ-7 scale (*p* ≤ 0.002, R = 0.53). The greatest impact was observed in the domains of emotional health and physical activity. The more incontinence symptoms reported by the respondent in the UDI-6 scale, the worse was her sexual satisfaction in the FSDS-R (*p* = 0.003, R = 0.39). UI in women contributes to the development of sexual dysfunctions, including decreased interest in sexual life, limited intercourse, and dissatisfaction with sexual life.

## 1. Introduction

Urinary incontinence (UI), due to the intimate nature of symptoms and their inconvenience in everyday life, is a cause of frustration in relation to women’s social, psychological and sexual needs [1,2,3]. According to the definition of the International Continence Society (ICS), urinary incontinence denotes the uncontrolled, involuntary flow of urine from the bladder [4,5,6,7]. The severity of urinary incontinence symptoms can range from passing small amounts of urine during coughing or sneezing to losing urine while sitting, walking or going downstairs and ultimately being unable to hold urine with a full bladder [8].

Feelings most often experienced by people afflicted with urinary incontinence include shame, helplessness, anxiety, reduced self-esteem and reduced social value. Many sufferers encounter difficulties in coping with UI and its resultant problems, and, in consequence, withdraw from customary physical and social activities, which in extreme situations can lead to complete isolation and predispose them to depression or other mental disorders. Urinary incontinence is the direct cause of a decline in the quality of intimate, personal, social and professional life [9,10].

One particular negative consequence of urinary incontinence among women is a reduction in or even complete abandonment of sexual activity [11]. The World Health Organization (WHO) defines sexual health as a state of physical, mental, emotional and social well-being related to a person’s sexuality, while sexual dysfunctions are classified as disorders that prevent an individual from participating in sexual relations in the desired manner. The severity of sexual dysfunctions may vary, ranging from minor symptoms, close to the norm, to strong symptoms, which completely impede the sexual activity of a given individual [12,13]. Female sexual dysfunctions constitute the sexual interest/arousal disorder (FSIAD), genito-pelvic pain/penetration disorder (GPPPD) and female orgasmic disorder (FOD). In the newest study on a large Polish group, 40% of women had sexual disorders, independent of medical disorders. In this report 17.1% of women reported lubrication problems, which was lower than in other population studies. The prevalence of orgasmic disorders was between 21 and 28.5%, which represents the average in many populations (10–34%, Europe and Asia). The prevalence of dyspareunia and vaginismus in the Polish population is also in the average range, as described in the literature [14].

Urinary incontinence and pelvic floor disorders can be a direct or indirect cause of sexual dysfunction; the result of which, in the most extreme cases, can be complete abandonment of sexual activity. In the study by Jha and Gopinath, nearly 36% of women with stress UI avoid sexual intercourse and 53% state that stress UI interferes with their sexual activity [13]. Urinary incontinence during intercourse (CI—coital incontinence) affects up to 40% of women with stress UI and 21% of women with pelvic organ prolapse. This is the most disturbing and most frequently reported problem in women who give up sexual contact [15]. However, successful UI treatment can improve sexual satisfaction [16]. 

Pelvic floor dysfunction (PFD) has a negative impact on the overall quality of life in 66% of women struggling with this problem, and 25% of patients report a deterioration in the quality of sexual functioning, which also manifests itself as a decline in the overall quality of life [12]. Sexual problems in people with PFD are most often manifested as disruption of the sex drive, dyspareunia, disturbance of the feeling of orgasm, leakage of urine during immission or orgasm, prolongation of the initial phase of intercourse, and limiting of the immission phase to the time necessary for the partner’s fulfillment [16,17,18]. During sexual activity, UI usually occurs during the insertion of the penis into the vagina in the case of stress urinary incontinence, and during orgasm in the case of overactive bladder [19,20].

The aim of this study is to obtain information on the subjective symptoms’ severity, the level of sexual dissatisfaction, and, finally, on the quality of life in the population of women suffering from urinary incontinence.

## 2. Materials and Methods

The current study was conducted on a group of 145 women who declared constant sexual activity and were diagnosed with UI by a gynecologist or urologist, accordingly, to the ICD-10 classification (N39.3). Thirteen patients were excluded from the study due to incomplete answers to questionnaires or non-fulfillment of the inclusion criteria. The study was carried out among the patients of the Department of Obstetrics and Gynecology in the Boni Fratres Catoviensis Hospital and of the Urological, Gynecological and Proctological Physiotherapy Center UROSILESIA in Zabrze. Before the study, patients were informed of its confidentiality and were presented with the research project’s aims and assumptions. Medical data were collected from January to March 2019.

Women who met the following inclusion criteria were eligible for the study: female gender, UI diagnosed by a urologist or gynecologist, current sexual activity, no prior surgical treatment of urinary incontinence. Women were excluded from the study if they met any of the criteria as follows: pre-existing chronic diseases, minor pelvic trauma, previous gynecological, urological or lumbosacral spine surgeries, present lower UTIs (urinary tract infections), and pelvic floor dysfunction in anterior compartment or apical POPQ (pelvic organ prolapse quantification) grade ≥ 2. The ICD-10 classification was used for preselection of subjects and mixed or urge urinary incontinence was excluded by the UDI-6 questionnaire.

The study was carried out using the diagnostic survey method with an authorial survey questionnaire and validated questionnaires: the FSDS-R (Female Sexual Distress Scale -Revised), the IIQ-7 (Incontinence Impact Questionnaire) and the UDI-6 (Urogenital Distress Inventory); Cronbach’s alpha results for Polish versions were previously assessed in the literature, equaling 0.86–0.92; 0.89 and 0.95, respectively [21,22]. The authorial survey questionnaire was divided into two parts, the first eliciting sociodemographic data on the respondents (i.e., age, body mass, height, place of residence, marital status, and work-experience information), and the second consisting of questions strictly concerned with the symptoms of UI, childbirth history and the respondent’s sexual activity. The FSDS-R is a scale of sexual dissatisfaction in women, based on their feelings and concerns from the previous four weeks. When filling out the questionnaire, respondents specify how often they have experienced such concerns by placing a number to the left of each item, where 0 means “never”, and 4 “always”. The maximum possible score is 52 points. However, any score greater than or equal to 13 is regarded as indicating sexual dissatisfaction. The higher the score, the lower the level of satisfaction with sexual life [21]. The UDI-6 is a questionnaire concerning symptoms of urinary incontinence [22,23], while the IIQ-7 assesses the quality of life in people with UI [22,23,24]. Overall results from both questionnaires range from 0 to 100. The results obtained from the Polish population indicate that a score of more than 33.33 points on the UDI-6 questionnaire shows higher suffering from UI symptoms. Regarding the IIQ-7 questionnaire: obtaining a result of between 0 and 50 shows a good quality of life; between 50 and 70 indicates moderate, and more than 70 shows a poor quality of life. The Bioethics Commission of the Medical University of Silesia in Katowice declared that the study presented here is not a medical experiment, and, therefore, evaluation by the Bioethics Commission was not required (KNW/0022/KB/38/19).

The data obtained in the course of this study were subjected to statistical analysis in a search for correlations between variables, which would allow the assessment of the quality of the sexual life of the respondents. The results were collected on a Microsoft Office Excel 2012 spreadsheet (Microsoft, Redmont, Washington, DC, USA), while the analysis and calculations were performed using the Statistica 12 PL StatSoft program (Cracow, Poland). The distribution of data was assessed using the Shapiro–Wilk test. Analysis of unrelated qualitative variables was performed using the chi-squared test. Intercorrelation scores were calculated using Pearson’s r coefficient. The Student’s *t*-test was used to analyze quantitative variables meeting the criteria of normal distribution. Throughout the study, the level of significance was set at *p* < 0.05.

## 3. Results

The study involved 145 respondents and the descriptive characteristics of the respondents are presented in Table 1.

Demographic characteristics were as follows: 41.2% lived in large cities with over 100.000 inhabitants, the second largest group (31.8%) were women from smaller towns (up to 50.000 inhabitants), while 27.0% lived in rural areas. A total of 51.7% of respondents had higher education, 29.23% had secondary education, and 19.1% had primary education. Over half of the study population (58.1%) were married, 19.8% were single, 17.9% were living in non-formalized intimate relationships, and 4.2% were widowed. Over half of the respondents (53.9%) declared a physical type of work, 28.2% performed intellectual work, and 17.9% were unemployed.

The percentage of respondents treated for urinary incontinence, and type of therapy, were also checked. A total of 57.3% of respondents declared no type of UI treatment, 38.9% of women declared regular appointments with a physiotherapist as first-line treatment, and 3.8% stated the use of pharmacological methods.

Changes in the sexual activity of women with urinary incontinence were analyzed. For 49.1% of women, the frequency of sexual activity before the onset of the UI episodes was greater than at present, 38.3% of women stated that their level of activity remained the same, and according to 12.6% of women, the frequency of their sexual activity was previously lower than at present, i.e., had increased. 

For a large subset of the women surveyed, the reason given for limiting sexual activity was fear of involuntary leakage of urine during intercourse—39.1%. However, 30.6% of women did not express any concern about this issue, and the remaining 30.3% of women did not experience this symptom.

Analysis of the results of the FSDS-R questionnaire, based on the answer key supplied, allowed assessment of the degree of sexual dissatisfaction among women. When considering the responses obtained, two groups of respondents could be distinguished: a group of women dissatisfied with their sex life (FSDS-R ≥ 13), consisting of 121 women (83.45%), and a group of women satisfied with their sex life (FSDS-R < 13) consisting of 24 women (16.55%). 

Table 2 shows the distribution of responses on the Female Sexual Distress Scale-Revised (FSDS-R). The women, most frequently, selected 0, 1 or 2, indicating little or medium sex-related distress for the majority of items. The median score for all participants was 29.00. They were least likely to profess feelings of “regrets about sexuality” ($\bar{x}$ = 1.90 ± 1.06) or of being “angry about their sex life” ($\bar{x}$ = 1.90 ± 0.09) and “stressed about sex” ($\bar{x}$ = 1.87 ± 1.03). They most often declared feeling “distressed about sex life” ($\bar{x}$ = 2.51 ± 1.20) and being “dissatisfied with sex life” ($\bar{x}$ = 2.36 ± 1.11).

The average results obtained from the questionnaires FSDS-R, UDI-6 and IIQ-7, together with descriptive statistics, are presented in Table 3.

Correlations between respondents’ ages, BMIs, and work experience, and results obtained from the UDI-6, IIQ-7 and FSDS-R questionnaires were analyzed. It was assumed that the value of *r* between 0.0 to 0.4, 0.4 to 0.7, and 0.7 to 1.0 was considered to show, respectively, a weak, moderate, and strong correlation. Among the statistically significant results (*p* < 0.05), the following relationships were found, as presented in Table 4.

Pregnancy constituted the main risk factor for the development of UI. The relationship between delivery history (number of pregnancies, births, vaginal deliveries, and cesarean sections) and respondents’ satisfaction or dissatisfaction with sexual life is presented in Table 5. As is shown, respondents after pregnancy and with a higher number of births were more often dissatisfied with their sexual lives (FSDS-R ≥ 13).

The results obtained from the IIQ-7 questionnaire, assessing the impact of UI on quality of life, were divided into four domains: physical activity, travel, social/relationships, and emotional health. Student’s *t*-test was used to verify the differences within each of the four IIQ-7 domains, between the two groups of women identified by the FSDS-R questionnaire. Statistically significant differences were obtained in the domains of physical activity (*p* = 0.007), social/relationship (*p* < 0.001) and emotional health (*p* = 0.004). Women dissatisfied with their sexual lives, according to the FSDS-R questionnaire, reported worse quality of life in the domains of physical activity, social/relationship and emotional health, according to the IIQ-7 questionnaire. See Table 6.

A positive correlation, calculated using Pearson’s r coefficient, was observed between scores obtained on the FSDS-R scale and scores on the IIQ-7 scale (*p* < 0.002, R = 0.53). The higher the score on the FSDS-R scale (i.e., higher level of dissatisfaction with sexual life), the lower was the quality of life in women with urinary incontinence.

A positive correlation was also observed between the UDI-6 and FSDS-R questionnaires, indicating that the higher the score in the UDI-6 questionnaire, the higher the score in the FSDS-R questionnaire, thereby indicating a low level of satisfaction with sexual life among respondents (*p* = 0.003, R = 0.39—Pearson’s r coefficient).

The UDI-6 and IIQ-7 questionnaires were analyzed using the Student’s t-test. It was found that the higher the score obtained by women in these questionnaires, the lower their sexual satisfaction. For instance, the UDI-6 questionnaire score for women with lower sexual satisfaction (FSDS-R ≥ 13) was 56.10 ± 18.89, and for women with higher sexual satisfaction (FSDS-R < 13) it was 40.96 ± 17.24. The differences in these UDI-6 scores were, however, not statistically significant. Turning to the IIQ-7 questionnaire, the score for the group of women with lower sexual satisfaction (FSDS-R ≥ 13) was equal to 63.15 ± 24.85, while in the group of women with higher sexual satisfaction (FSDS-R < 13) the score was 17.26 ± 11.89. These differences were statistically significant. See Table 7.

## 4. Discussion

Women’s sexuality is becoming more frequently analyzed through the prism of various sexual dysfunctions and disorders that negatively affect quality of life. Urinary leakage during an intimate situation is a more common problem than may be expected; indeed, incontinence can occur in sexually active women at any age.

Urinary incontinence during intercourse is an embarrassing situation for both partners. However, for a woman it can be a traumatic experience, often leading to sexual dysfunction and even abandonment of sexual activity. During an intimate situation, there is additional pressure on the lower abdomen which may lead to uncontrolled urination. Women who have had such an experience often avoid intimacy with their partners because their self-confidence decreases and they feel they are lacking in control over their own bodies. Reported incidence of CI among women with UI varies between 10% and 27%. Therefore, assessing the influence of incontinence on sexual functioning is of particular importance [25].

The analysis undertaken in this study, of changes in the sexual activity of women with urinary incontinence, as compared to the period before the onset of symptoms, shows that 49.1% of respondents reported a deterioration in sexual relations, which was mostly caused by fear of involuntary urine leakage during intercourse. Based on a five-year observation of a group of 1256 mature women, Ugurlucan et al. drew similar conclusions [26]. Their results showed that urinary incontinence during intercourse had a negative impact on the sexual functioning and quality of life of the women observed. Their results were obtained using the KHQ (King’s Health Questionnaire), FSFI (Female Sexual Function Index), and PISQ-12 (Pelvic Organ Prolapse/Urinary Incontinence Sexual Function Questionnaire). The data from the FSFI questionnaire showed that the most disturbed domains were desire and arousal. In turn, Sarikaya et al., in their survey of studies based on the FSFI and FSDS questionnaires, found increased rates of sexual dysfunction in both genders. Moreover, a higher risk of depression was observed among patients with urinary incontinence, which was assessed using the Beck Depression Scale [27]. As the UI has multifactorial influences, the menopausal status should also be discussed. Epidemiological studies in a wider age range, such as that in our study, have shown an initial peak of UI in perimenopause (age 45–54) followed by another increase that begin at age of 70 [28]. In a study with a range of subjects similar to our group, by Nagai et al. (age 27–82), the overall prevalence of UI was 19.3% [29]. But even with accurate estimation of menopause timing in longitudinal follow-up studies, these are insufficient to separate the effect of concurrent age-related conditions from the potential contribution of the menopause [28].

In this study, the group of women dissatisfied with their sex life, according to the FSDS-R, constitutes 83.45% of respondents, and the deterioration in their quality of life is most evident in the areas of emotional health, social/relationship and physical activity, according to the IIQ-7 questionnaire. Cameron et al. conducted a study on a group of 510 women with LUTS (lower urinary tract symptoms), of which 420 respondents had UI symptoms [30]. Their study was based on the Pelvic Floor Distress Inventory (PFDI-20) and Pelvic Organ Prolapse/Incontinence Sexual Questionnaire (PISQ-IR). Their results allowed them to conclude that sexually active women with UI obtained significantly lower results in the PISQ-IR, which manifests itself in lower sexual satisfaction (*p* < 0.01). It was also noted that respondents with mixed UI (N = 240) showed a lower quality of sexual life compared to patients with stress UI and urgency UI. In a study using the UDI-6 and IIQ-7 questionnaires, Zilberlicht et al. showed that urinary incontinence in women affects their sexual functioning (r = 0.42, *p* = 0.031), which in turn leads to a deterioration in their quality of life [31].

The results from the UDI-6 and FSDS-R questionnaires and their correlations, obtained in this study, show that there is a severity-dependent relationship between incontinence and quality of sexual life among the women surveyed. The greater the urine leakage, the lower the quality of sexual life. A meta-analysis by Duralde et al. shows that the severity of incontinence in women is associated with increased rates of sexual dysfunction, with urinary incontinence during intercourse affecting between 24% and 66% of all women included in the research studies [32]. A study by Felippe et al. evaluating the impact of urinary incontinence on sexual dysfunction showed that women with urinary incontinence are more prone to sexual abstinence than women without this problem [33]. In addition, women with urinary incontinence show lower sexual desire and worse sexual satisfaction than women without UI, despite a similar frequency of sexual activity. The worst results in terms of sexual functioning (according to SQ-F—Sexual Quotient-Female) were presented by women with the most severe problems of urinary incontinence. We assumed that years of work experience and a physical type of work could influence the UI, but the correlations were observed in relation to sexual activity, rather than to UI itself. This observation has limited power, because we did not assess exact physical expenditure in order to correlate UI with sexual life, such as has been performed in many studies among athletes [6].

The study presented here has the advantage of having used three standardized questionnaires: UDI-6, IIQ-7, and FSDS-R. However, a significant limitation in this study is the small number of women who participated in it. This is due to the fact that women with the problems of urinary incontinence and sexual dysfunction are generally reluctant to consult a specialist. Other limitations include no precise data on hormonal status, physical expenditure at work, patient recruitment in an ambulatory office, declarative data from UDI-6 questionnaire and the heterogeneity of the study group in many aspects (current therapy, work, menopause status etc.). It seems likely that expanding this survey to other groups of respondents would further highlight the scale of the problem of deterioration of sexual life among women with urinary incontinence.

## 5. Conclusions

Leakage of urine in women during sexual intercourse contributes to the development of sexual dysfunctions, reducing interest in sexual life. Urinary incontinence in women reduces frequency of intercourse compared to the period before the onset of the symptoms of incontinence. UI increases dissatisfaction with sexual life and intensifies fear of sexual contact.

We consider that the present paper indicates the importance of the implementation of effective methods for UI prevention and the management of urinary incontinence symptoms (i.e., physiotherapy, pharmacotherapy, surgery). These factors, along with education concerning methods of safe sexual intercourse while suffering from UI, give healthcare professionals the instruments for generating improvement in the quality of sexual health in women with UI.

## Figures and Tables

**Table 1:** Characteristics of the study group.

Characteristic	$\bar{x}$ ± SD	Minimum	Maximum	Median	Q_1_–Q_3_
Age (years)	46 ± 17.60	21	72	47	35–58
BMI (kg/m^2^)	24.93 ± 3.92	18.06	36.92	23.53	23–29
Work experience (years)	24 ± 12.24	1	38	25	3–20

$\bar{x}$—mean, SD—Standard deviation, BMI—Body mass index, Q_1_–Q_3–_lower quartile-upper quartile.

**Table 2:** Distribution of responses to the Female Sexual Distress Scale-Revised (FSDS-R) items (N = 145).

FSDS-R Item	Never		Rarely		Occasionally		Frequently		Always		$\bar{x}$ ± SD
	N	%	N	%	N	%	N	%	N	%	
1. Distressed about sex life	9	6.2	18	12.4	49	33.8	28	19.3	41	28.3	2.51 ± 1.20
2. Unhappy about sexual relationship	6	4.1	27	18.6	38	26.2	62	42.8	12	8.3	2.32 ± 1.00
3. Guilty about sexual difficulties	12	8.3	26	17.9	41	28.3	45	31.0	21	14.5	2.25 ± 1.15
4. Frustrated by sexual problems	9	6.2	29	20.0	49	33.8	37	25.5	21	14.5	2.22 ± 1.11
5. Stressed about sex	18	12.4	24	16.5	70	48.4	24	16.5	9	6.2	1.87 ± 1.03
6. Inferior because of sexual problems	18	12.4	32	22.1	44	30.3	33	22.8	18	12.4	2.00 ± 1.20
7. Worried about sex	18	12.4	12	8.3	67	46.2	27	18.6	21	14.5	2.14 ± 1.15
8. Sexually inadequate	18	12.4	12	8.3	66	45.5	37	25.5	12	8.3	2.08 ± 1.07
9. Regrets about sexuality	15	10.4	35	24.1	53	36.5	33	22.8	9	6.2	1.90 ± 1.06
10. Embarrassed about sexual problems	21	14.5	9	6.2	41	28.3	56	38.6	18	12.4	2.28 ± 1.20
11. Dissatisfied with sex life	12	8.3	24	16.5	20	13.8	77	53.1	12	8.3	2.36 ± 1.11
12. Angry about sex life	19	13.1	9	6.2	90	62.1	21	14.5	6	4.1	1.90 ± 0.09
13. Bothered by low sexual desire	24	16.5	26	17.9	46	31.8	28	19.3	21	14.5	1.97 ± 1.27

FSDS-R—Female Sexual Distress Scale-Revised, N—Number of participants, $\bar{x}$—mean, SD—Standard deviation.

**Table 3:** Descriptive statistics of the results obtained from questionnaires (N = 145).

Questionnaire	$\bar{x}$ ± SD	Minimum	Maximum	Median	Q_1_–Q_3_
FSDS-R	27.85 ± 12.31	1.00	52.00	29.00	7.5–38
UDI-6	53.60 ± 19.41	11.00	99.99	50.00	18–55
IIQ-7	55.56 ± 28.81	0.00	99.99	66.66	16–64

$\bar{x}$—mean, SD—Standard deviation, Q_1_-Q_3_—lower quartile-upper quartile, FSDS-R—Female Sexual Distress Scale-Revised, UDI-6—Urogenital Distress Inventory, IIQ-7—Incontinence Impact Questionnaire.

**Table 4:** Correlations between the results obtained from the questionnaires and the data characterizing the studied group (N = 145).

Characteristic	UDI-6	IIQ-7	FSDS-R
Age (years)	r = 0.0644 *p* = 0.441	r = 0.1836 *p* = 0.027 *	r = 0.1528 *p* = 0.067
BMI (kg/m^2^)	r = 0.1213 *p* = 0.146	r = 0.3148 *p* < 0.001 *	r = 0.0428 *p* = 0.609
Work experience (years)	r = 0.0426 *p* = 0.611	r = 0.2566 *p* = 0.002 *	r = −0.1935 *p* = 0.002 *

FSDS-R—Female Sexual Distress Scale-Revised, UDI-6—Urogenital Distress Inventory, IIQ-7—Incontinence Impact Questionnaire, BMI—Body mass index, r—correlation coefficient, *p*—*p*-value, *—*p* < 0.05 (Pearson correlation *r* test, *p* < 0.05).

**Table 5:** Analysis of the influence of delivery history on the level of satisfaction with sexual life, according to the FSDS-R scale (N = 145).

Delivery History	FSDS-R < 13 N = 24 (16.55%) $\bar{x}$ ± SD	FSDS-R ≥ 13 N = 121 (83.45%) $\bar{x}$ ± SD	*p*
Number of pregnancies	2.57 ± 1.43	2.08 ± 0.82	0.0005 *
Number of births	1.85 ± 1.01	1.83 ± 0.81	0.0147 *
Number of vaginal deliveries	1.42 ± 0.92	1.47 ± 0.88	0.7200

FSDS-R—Female Sexual Distress Scale-Revised, N—Number of participants, $\bar{x}$—mean, SD—Standard deviation, *p*—*p*-value, *—*p* < 0.05 (Student’s *t*-test, *p* < 0.05).

**Table 6:** Analysis of the IIQ-7 domains for two groups of women—satisfied with their sex life (FSDS-R < 13) and dissatisfied with their sex life (FSDS-R ≥ 13).

IIQ-7 Domain	FSDS-R < 13 N = 24 (16.55%) $\bar{x}$ ± SD	FSDS-R ≥ 13 N = 121 (83.45%) $\bar{x}$ ± SD	*p*
Physical activity	14.58 ± 13.78	52.19 ± 26.07	0.007 *
Travel	12.49 ± 11.25	55.09 ± 32.18	0.085
Social/relationships	18.49 ± 16.48	69.69 ± 35.22	<0.001 *
Emotional health	27.08 ± 20.74	78.91 ± 21.91	0.004 *

IIQ-7—Incontinence Impact Questionnaire, FSDS-R—Female Sexual Distress Scale-Revised, N—Number of participants, $\bar{x}$—mean, SD—Standard deviation, *p*—*p*-value, *—*p* < 0.05 (Student’s *t*-test, *p* < 0.05).

**Table 7:** Analysis of the UDI-6 and IIQ-7 questionnaires scores in two groups of women—FSDS-R < 13 and FSDS-R ≥ 13.

Questionnaire	FSDS-R < 13 N = 24 (16.55%) $\bar{x}$ ± SD	FSDS-R ≥ 13 N = 121 (83.45%) $\bar{x}$ ± SD	*p*
UDI-6	40.96 ± 17.24	56.10 ± 18.89	0.000 *
IIQ-7	17.26 ± 11.89	63.15 ± 24.85	0.001 *

UDI-6—Urogenital Distress Inventory, IIQ-7—Incontinence Impact Questionnaire, FSDS-R—Female Sexual Distress Scale-Revised, N—Number of participants, $\bar{x}$—mean, SD—Standard deviation, *p*—*p*-value, *—*p* < 0.05 (Student’s *t*-test, *p* < 0.05).

## Data Availability

The data underlying this article will be shared upon reasonable request to the corresponding author.
